# Calcium Co-regulates Oxidative Metabolism and ATP Synthase-dependent Respiration in Pancreatic Beta Cells

**DOI:** 10.1074/jbc.M113.513184

**Published:** 2014-02-19

**Authors:** Umberto De Marchi, Jonathan Thevenet, Aurelie Hermant, Elhadji Dioum, Andreas Wiederkehr

**Affiliations:** From the ‡ Mitochondrial Function and; § Diabetes and Metabolic Health, Nestlé Institute of Health Sciences, 1015 Lausanne, Switzerland

**Keywords:** Calcium, Energy Metabolism, Insulin, Islet, Mitochondria, NAD

## Abstract

Mitochondrial energy metabolism is essential for glucose-induced calcium signaling and, therefore, insulin granule exocytosis in pancreatic beta cells. Calcium signals are sensed by mitochondria acting in concert with mitochondrial substrates for the full activation of the organelle. Here we have studied glucose-induced calcium signaling and energy metabolism in INS-1E insulinoma cells and human islet beta cells. In insulin secreting cells a surprisingly large fraction of total respiration under resting conditions is ATP synthase-independent. We observe that ATP synthase-dependent respiration is markedly increased after glucose stimulation. Glucose also causes a very rapid elevation of oxidative metabolism as was followed by NAD(P)H autofluorescence. However, neither the rate of the glucose-induced increase nor the new steady-state NAD(P)H levels are significantly affected by calcium. Our findings challenge the current view, which has focused mainly on calcium-sensitive dehydrogenases as the target for the activation of mitochondrial energy metabolism. We propose a model of tight calcium-dependent regulation of oxidative metabolism and ATP synthase-dependent respiration in beta cell mitochondria. Coordinated activation of matrix dehydrogenases and respiratory chain activity by calcium allows the respiratory rate to change severalfold with only small or no alterations of the NAD(P)H/NAD(P)^+^ ratio.

## Teaser

**Background:** Nutrients stimulate calcium dependent activation of energy metabolism, in pancreatic beta cells.

**Results:** Glucose-induced ATP synthase-dependent respiration is strictly calcium-dependent, with little or no effect of calcium on the NAD(P)H response.

**Conclusion:** Calcium coordinates oxidative metabolism and respiration in pancreatic beta cells.

**Significance:** Calcium has novel mitochondrial targets downstream of mitochondrial dehydrogenases.

## Introduction

Cellular energy demand and mitochondrial activation are linked and tightly regulated, which is required for normal energy homeostasis. In aerobic tissues the fraction of glycolytic ATP synthesis is relatively modest (<10%), and mitochondria are, therefore, crucial to maintain a high ATP/ADP P_i_ ratio (phosphorylation potential) to drive energy consuming cellular processes. An increased energy demand, as observed during stimulation of a cell, enhances ATP hydrolysis that must be compensated for by accelerated ATP synthesis. For example in the heart, mitochondrial respiration can adjust over a wide range matching the enhanced work load associated with an increase in beating frequency. Interestingly, energy intermediates such as ATP, ADP, and phosphocreatine remain unchanged even when the heart frequency is augmented severalfold (1). Similarly, activation of specific brain areas augments their demand for ATP. This extra energy need is covered almost exclusively by elevated mitochondrial ATP synthesis with little contribution of glycolysis (2). Likewise, in the insulin-secreting pancreatic beta cell, energy demand and mitochondrial activation are closely intertwined. In response to glucose, beta cell mitochondria are able to augment their oxidative metabolism (3), respiration (4, 5), and mitochondrial ATP synthesis rates (6) to match cytosolic energy consuming processes. As a consequence, nutrient stimulation raises the cytosolic ATP/ADP P_i_ ratio (4, 7, 8). This increase is maintained despite enhanced ATP consumption due to beta cell electrical activity, insulin secretion, biosynthesis, and transport. These examples illustrate the potential of mitochondria to adjust their activity to energy demand without any prior energy stress such as a drop of the phosphorylation potential.

In pancreatic beta cells, the rise in the cytosolic ATP/ADP P_i_ ratio after nutrient stimulation is necessary to initiate plasma membrane electrical activity. ATP promotes closure of the ATP-sensitive potassium channel (*K*_ATP_),^3^ which will depolarize the beta cell plasma membrane. As a result, voltage-dependent Ca^2+^ channels open, and associated cytosolic Ca^2+^ rises trigger insulin granule exocytosis.

Mitochondrial ATP synthesis is driven by the electrochemical gradient across the inner mitochondrial membrane, which is established by the proton extruding activity of complex I, III, and IV of the respiratory chain. Re-entry of protons through complex V (ATP synthase) is coupled to the synthesis of ATP from ADP and inorganic phosphate (P_i_). Both the electrical and the chemical proton gradient across the inner mitochondrial membrane are elevated after stimulation of beta cells with nutrients (6, 9). This increase in driving force in part explains accelerated mitochondrial ATP synthesis. In addition, specific signals inside the mitochondrial matrix control the kinetics of ATP synthesis (5, 6, 10, 11). Ca^2+^ is an important activator of mitochondrial energy metabolism (5, 11–13). Stimulation of oxidative metabolism by mitochondrial Ca^2+^ is required to maintain a normal NAD(P)H/NAD(P)^+^ ratio as demonstrated in neurons and cardiomyocytes (14, 15). In beta cells the mitochondrial Ca^2+^ signals closely follow cytosolic Ca^2+^ transients (16). Preventing Ca^2+^ signaling in beta cells reduces glucose-induced respiration (5, 17), whereas hyperpolarization of the mitochondrial inner membrane potential is normal (6). Specific buffering or suppression of mitochondrial Ca^2+^ rises lowers glucose-induced respiration and ATP synthesis and impairs second phase insulin secretion (5, 11). These data have established mitochondrial Ca^2+^ as a critical signal for the activation of mitochondrial energy metabolism.

This Ca^2+^ effect has been proposed to be the result of activating matrix dehydrogenases such as α-ketoglutarate dehydrogenase, isocitrate dehydrogenases, and pyruvate dehydrogenase (13). These dehydrogenases form the NADH, which provides electrons to complex I of the respiratory chain. Whether the activation of dehydrogenases by mitochondrial Ca^2+^ is relevant for beta cell function is not clear. Preventing glucose-induced Ca^2+^ influx does not appear to reduce the NAD(P)H response to glucose in this cell type (17, 18). In addition, regulation of pyruvate dehydrogenase by Ca^2+^ does not affect insulin secretion (19, 20). It is likely that alternative mitochondrial Ca^2+^ targets are able to activate mitochondrial energy metabolism. Several such additional mitochondrial Ca^2+^ targets have been proposed (21), but for most part they remain elusive.

Here we have studied the mechanism resulting in enhanced energy metabolism in pancreatic beta cells during glucose stimulation. We observe a pronounced glucose-induced activation of ATP synthase-dependent respiration in INS-1E insulinoma cells and human islets. Activation of ATP synthase-dependent respiration but not the NAD(P)H response is potentiated by Ca^2+^. We propose that coordinated activation of oxidative metabolism and respiration allows Ca^2+^ to accelerate mitochondrial respiration and ATP synthesis without significantly affecting steady-state NAD(P)H levels.

## EXPERIMENTAL PROCEDURES

### 

#### 

##### Reagents

Chemicals were from Sigma, Invitrogen, or VWR unless otherwise indicated. The YC3.6_cyto_ pcDNA3 (22) and 4mtD3cpv pcDNA3 constructs (23) were kindly provided by Dr. A. Miyawaki (Riken Brain Science Institute, Wako, Japan) and Dr. R. Tsien (University of California, San Diego), respectively. The adenovirus expressing YC3.6_cyto_ under the control of the rat insulin promoter (Ad-RIP-YC3.6_cyto_) was a kind gift of Prof. C. B. Wollheim (University of Geneva).

##### INS-1E Cell Culture

INS-1E cells were cultured at 37 °C in a humidified atmosphere (5% CO_2_) in RPMI 1640 medium (Invitrogen) containing 11 mm glucose, supplemented with 10 mm Hepes (pH 7.3), 10% (v/v) heat-inactivated fetal calf serum (FCS; Brunschwig AG), 1 mm sodium pyruvate, 50 μm β-mercaptoethanol, 50 μg/ml penicillin, and 100 μg/ml streptomycin.

##### Human Islet

Human islets from non-diabetic deceased donors were purchased from Tebu-bio. Donors had consented to donate organs for medical research. The islets were cultured at 37 °C in a humidified atmosphere (5% CO_2_) in RPMI 1640 medium complemented with 5.55 mm glucose, 10% (v/v) FCS (Brunschwig AG), 10 mm HEPES (pH 7.3), 1 mm sodium pyruvate, 50 μm β-mercaptoethanol, 50 μg/ml penicillin, 100 μg/ml streptomycin, and 50 μg/ml gentamycin (islet medium).

##### 804G Matrix

The 804G bladder carcinoma cell line (24) and protocols to isolate the matrix were obtained from Viacyte (San Diego, CA). 804G cells were cultured at 37 °C in a humidified atmosphere (5% CO_2_) in DMEM containing 5.55 mm glucose supplemented with 10% (v/v) FCS (Invitrogen), 50 μg/ml penicillin, and 100 μg/ml streptomycin. Once cells reached 80% confluency, the culture medium was changed to the same medium lacking serum. After 2 days, the culture medium containing the matrix was collected and stored at −20 °C. Glass coverslips or Seahorse tissue culture plates were coated overnight with 804G matrix.

##### Single Cell Imaging of Cytosolic and Mitochondrial Ca^2+^ Signals

Cytosolic or mitochondrial Ca^2+^ was measured with the genetically encoded cameleon sensors YC3.6_cyto_ or 4mtD3cpv. INS-1E cells were plated on polyornithine-treated 35-mm-diameter glass-bottom dishes (MatTek) and transfected with pcDNA3 vector carrying the Ca^2+^ sensor using Lipofectamine 2000 transfection reagent (Invitrogen). Two days after transfection cells were washed 4 times, and experiments were performed at 37 °C in Krebs-Ringer bicarbonate Hepes buffer (KRBH) containing 140 mm NaCl, 3.6 mm KCl, 0.5 mm NaH_2_PO_4_, 0.5 mm MgSO_4_, 1.5 mm CaCl_2_, 10 mm Hepes, and 5 mm NaHCO_3_ (pH 7.4). Glass coverslips were inserted in a thermostatic chamber (Life Imaging Services). The cells were preincubated for >15 min in KRBH basal (2.5 mm) glucose before recording. Cells were imaged on a DMI6000 B inverted fluorescence microscope using a HCX PL APO 63×/1.40–0.60 NA oil immersion objective (Leica Microsystems) and an Evolve 512 back-illuminated CCD with 16 × 16 pixels camera (Photometrics, Tucson, AZ). Cells were excited at 430 nm through a BP436/20 filter. The two emission images were acquired with BP480/40 and BP535/30 emission filters. Fluorescence ratios were calculated in MetaFluor 7.0 (Meta Imaging Series) and analyzed in Excel (Microsoft) and GraphPad Prism 5 (GraphPad). Images were taken every 2 s.

Human islets were infected for 90 min at 37 °C with Ad-RIP-YC3.6_cyto_. Experiments were performed 1–2 days later as described for INS1E-cells. The islets were incubated under resting conditions (KRBH 1 mm glucose) for >15 min before initiating the experiment.

##### Static Insulin Secretion from Human Islets

Islets were washed 3 times in KRBH containing 1 mm glucose. After 30 min of preincubation, in KRBH 1 mm glucose, the islets were either maintained in buffer containing 1 mm glucose, or the glucose concentration was raised to 16.7 mm for an additional 30 min. Insulin secretion is expressed as percent of content and was measured as described previously (10).

##### Oxygen Consumption Measurements

Oxygen consumption in INS-1E cells or human islets was measured using a XF96 instrument (Seahorse Biosciences). INS-1E cells were seeded into polyornithine-coated Seahorse tissue culture plates at a density of 20,000 cells per well. Two days later the cells were washed 2 times in KRBH 2.5 mm glucose. Human islets were bound to 804G matrix-coated plates. The islets were analyzed 24 h after plating. Before the measurement the human islets were washed twice in basal KRBH containing 1 mm glucose. Respiration rates were determined every 6 min. All experiments were performed at 37 °C. Respiratory chain inhibitors were added as indicated in the figures at the following concentrations: oligomycin (2.5 μg/ml), rotenone (1 μm), and antimycin A (1 μg/ml). ATP synthase-dependent respiration was calculated as the difference in respiration rate before and after the addition of oligomycin. Other mitochondrial respiration (ATP synthase independent) was obtained after subtracting oligomycin-dependent respiration from the total mitochondrial respiration, which corresponds to the difference in respiration rate before and after the addition of rotenone plus antimycin A. Total human islet protein was quantified using a bicinchoninic acid-based protein assay (Pierce, BCA protein assay kit no. 23225).

##### Cellular ADP and ATP

ADP and ATP were measured enzymatically as described (25). Cell extracts were prepared from INS-1E cells incubated for a total of 30 min in standard KRBH containing 1.5 mm Ca^2+^ or a KRBH lacking Ca^2+^ but including 0.4 mm EGTA. Where the contribution of ATP synthase was assessed, oligomycin (2.5 μg/ml) was added during the last 10 min of the 16.7 mm glucose incubation.

##### NAD(P)H Measurements

INS-1E cells or human islets were allowed to adhere to MatTek dishes coated with polyornithine or 804G matrix, respectively. All experiments were performed in KRBH buffer. A laser-scanning confocal microscope (Leica TCS SP5 II MP, Mannheim, Germany) with a HCX IRAPO L 25×/0.95 water objective was utilized to monitor NAD(P)H autofluorescence at 37 °C (Life Imaging Services).

Laser scans at 727 nm (IR Laser Chameleon ultra, Coherent) were used for two-photon excitation of NAD(P)H. Each 512 × 512 pixel image represents an average of 16 scans taken with a resonant scanner at 8000 Hz. The NAD(P)H emission band was collected at a 445–495-nm wavelength every 20 s. The excitation power of the laser was set to avoid cellular photo-damage. Data were processed with LAS AF software (Leica) and analyzed in Excel (Microsoft) and GraphPad Prism 5 (GraphPad). To normalize the NAD(P)H responses experiments were concluded by hydrogen peroxide addition, resulting in the almost complete loss of autofluorescence (minimal value). Data were normalized to both base line = 1 and a minimal value (hydrogen peroxide) = 0. Alternatively, rotenone was used for standardization. The complex I inhibitor increases the NAD(P)H signal to the point where autofluorescence is likely close to maximal. NAD(P)H responses were normalized as a fraction between basal (set to 0) and maximal signal after rotenone (set to 1). The change in fluorescence (Δ NAD(P)H) was calculated as the difference between base-line value and the effect of glucose after reaching a plateau. In addition, data were fitted during stimulation with a one-phase exponential association equation to extract τ_½_.

##### Statistical Analysis

Values are given as the mean ± S.E. *n* is the number of independent experiments. *p* values were obtained by Student's *t* test.

## RESULTS

### 

#### 

##### Inhibition of Mitochondrial ATP Synthesis Rapidly Blocks Glucose-induced Cytosolic and Mitochondrial Ca^2+^ Rises in Insulin Secreting Cells

**FIGURE 1.**
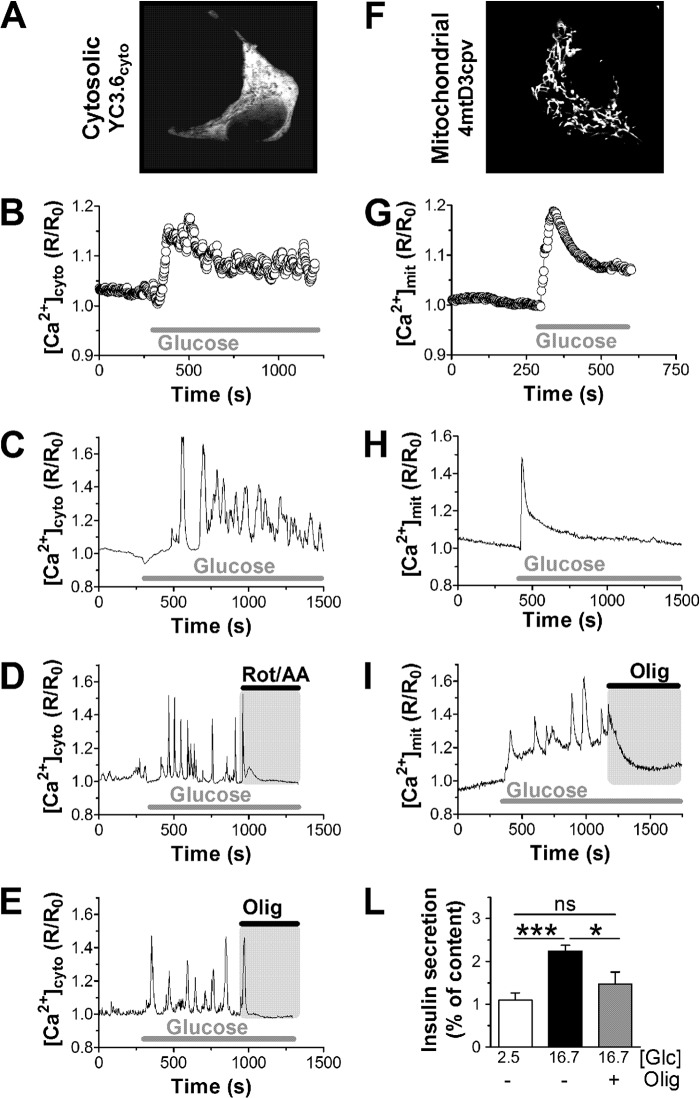
**Inhibition of mitochondrial ATP synthesis rapidly ends glucose-induced cytosolic and mitochondrial calcium signals.** Calcium signals were measured in INS-1E cells expressing the cytosolic Ca^2+^ probe YC3.6 (*A–E*) or the mitochondria-targeted Ca^2+^ sensor 4mtD3cpv (*F–I*). *A* and *E*, localization of the Ca^2+^ probe YC3.6 (*Cytosolic*; *A*) and 4mtD3cpv (*Mitochondrial*; *F*) recorded at 535 nm. The ratiometric signals were normalized to basal (set to 1). INS-1E cells were stimulated with 16.7 mm glucose as indicated. Average cytosolic (*B*; 116 cells, *n* = 11) and mitochondrial (*G*; 61 cells, *n* = 10) Ca^2+^ responses to glucose are presented. Examples of cytosolic (*C–E*) and mitochondrial (*H* and *I*) Ca^2+^ responses in individual INS-1E cells are also shown. *D*, respiration was blocked using rotenone (*Rot*; 1 μm) in combination with antimycin A (*AA*; 1 μg/ml). *E* and *I*, ATP synthase was inhibited with oligomycin (*Olig*; 2.5 μg/ml). Data are representative of 116 cells (*n* = 11) (*C*), 34 cells (*n* = 4) (*D*), and 27 cells (*n* = 3) (*E*). Mitochondrial traces (*H* and *I*) are representative of 61 cells (*n* = 10) and 24 cells (*n* = 4), respectively. *L*, static insulin secretion from INS-1E incubated for 30 min in the presence of resting (*white bar*) and stimulatory (*black* and *gray bar*) glucose (*Glc*) concentrations as indicated (in mm). Oligomycin (2.5 μg/ml) caused a reduction of glucose-stimulated insulin secretion (*gray bar*). Shown is the average ±S.E. from 6 measurements (*n* = 2) *, *p* < 0.05; ***, *p* < 0.0001; *ns*, not significant.

**FIGURE 2.**
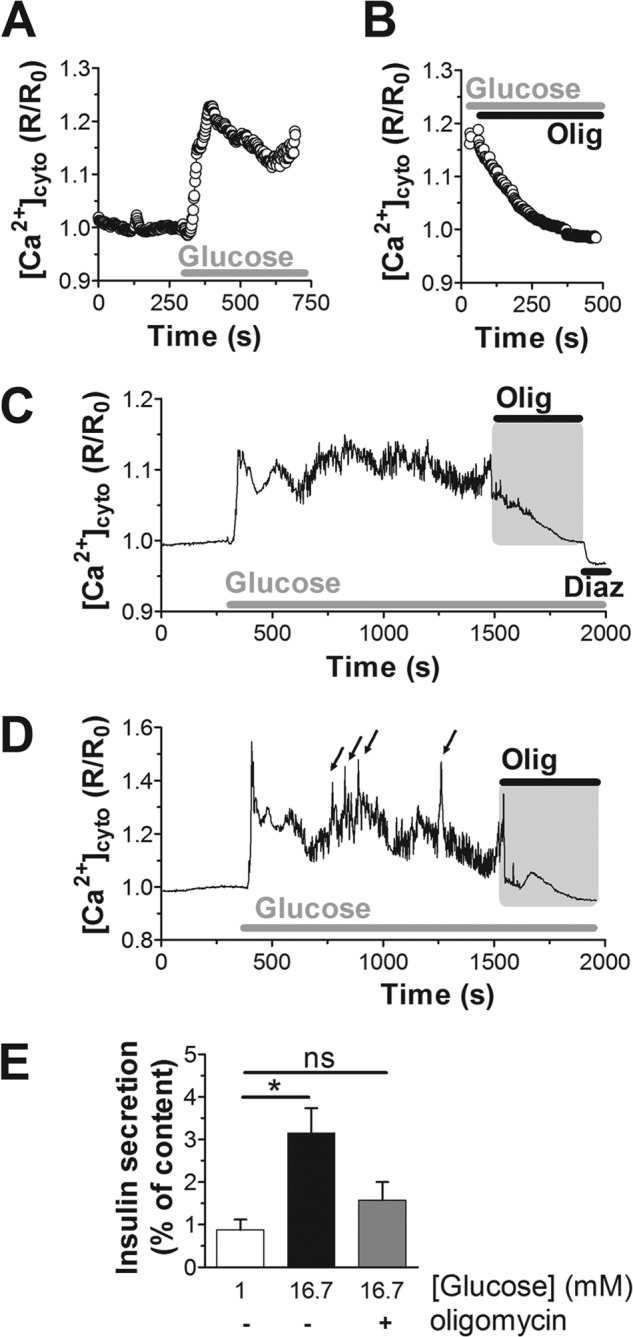
**Inhibition of ATP synthase terminates cytosolic calcium signals in human islet beta cells.** *A–D*, cytosolic Ca^2+^ responses were measured specifically in islet beta cells expressing YC3.6 under the control of the rat insulin promoter. The average Ca^2+^ response to 16.7 mm glucose (*A*; 40 cells, *n* = 5) and inhibition by oligomycin (*B*; *Olig*; 2.5 μg/ml; 40 cells, *n* = 5) are shown. The human islets analyzed were from two donors. *C* and *D*, Ca^2+^ measurements on individual beta cells in the context of the intact islet. *Arrows* indicate Ca^2+^ spikes superimposed on top of the net Ca^2+^ increase. Diazoxide (*Diaz*) was added at a final concentration of 100 μm. *E*, static insulin secretion from human islets (1 donor; *n* = 4). Islets were incubated in 1 mm glucose (*white bar*) or stimulated with 16.7 mm glucose (*black bar*). Oligomycin (2.5 μg/ml) prevented glucose-stimulated insulin secretion (*gray bar*). The difference between glucose stimulation with or without oligomycin did not reach significance (*p* = 0.07); *, *p* < 0.01; *ns*, not significant.

Glucose metabolism initiated cytosolic Ca^2+^ signals in INS-1E cells (Fig. 1, *A–E*) and human islet beta cells (Fig. 2, *A*, *C*, and *D*). In INS-1E cells the glucose-induced Ca^2+^ rise was preceded by a transient reduction of cytosolic Ca^2+^ followed by an initial Ca^2+^ overshoot before the cytosolic Ca^2+^ concentrations reached an apparent new steady state (Fig. 1*B*). At the single-cell levels the cytosolic Ca^2+^ responses to glucose were quite complex and variable (for examples see Fig. 1, *C–E*). At basal glucose concentrations, Ca^2+^ transients were usually absent or of small amplitude. After glucose (16.7 mm) stimulation, the amplitude and/or the frequency of the cytosolic Ca^2+^ signals increased (Fig. 1, *C–E*). In INS-1E and human islets it takes on average 73 ± 13 and 51 ± 14 s, respectively, before glucose-induced Ca^2+^ transients are initiated. Ca^2+^ transients depend on plasma membrane depolarization initiated by the closure of *K*_ATP_ channels. The *K*_ATP_ channel opener diazoxide, therefore, hyperpolarizes beta cells and lowered cytosolic Ca^2+^ below basal levels (Fig. 2*C*). Mitochondria act upstream of this process by controlling the cellular ATP/ADP ratio. Inhibition of respiration, therefore, blocks insulin secretion by interrupting the triggering pathway (26, 27). The combination of rotenone (complex I inhibitor) and antimycin A (complex III inhibitor) blocks respiration independent of the entry site of reducing equivalents into the respiratory chain. Inhibition of respiration in INS-1E cells caused a rapid loss of glucose-induced Ca^2+^ transients (Fig. 1*D*). The inhibition of Ca^2+^ transient was almost complete and very rapid (93.5 ± 75 s). Usually, only 1–2 more transients were observed after blocking respiration. Oligomycin blocked mitochondrial ATP synthesis but only partially inhibited respiration. Nevertheless, oligomycin arrested cytosolic Ca^2+^ transients over a similar time-course as rotenone and antimycin A (Fig. 1*E*).

Glucose-induced Ca^2+^ rises are translated into mitochondria. In the matrix Ca^2+^ signals can be studied using 4mtD3cpv, a calcium probe that is efficiently targeted to the mitochondria of INS-1E cells (Fig. 1*F*). Glucose rapidly induced a large mitochondrial Ca^2+^ transient before a new steady state was reached (Fig. 1*G*). At the single cell level the mitochondrial Ca^2+^ responses were quite variable (Fig. 1, *H* and *I*) but were frequently initiated by a larger mitochondrial Ca^2+^ transient (Fig. 1*H*). Preventing mitochondrial ATP synthesis using oligomycin ended mitochondrial Ca^2+^ spikes, and the matrix Ca^2+^ concentration returned to basal levels (Fig. 1*I*). Oligomycin, therefore, ended glucose-induced Ca^2+^ rises in both the cytosolic and mitochondrial compartment, causing a pronounced reduction of glucose-stimulated insulin secretion (Fig. 1*L*).

In human islets, blocking mitochondrial ATP synthesis using oligomycin had a similar effect on beta cell Ca^2+^ signaling (Fig. 2, *B-D*). The small cytosolic Ca^2+^ transients that can be observed at 16.7 mm glucose ceased shortly after blocking ATP synthesis (Fig. 2, *C* and *D*), and the net cytosolic Ca^2+^ concentrations returned to base line within minutes after inhibition of ATP synthase (285 ± 29 s). Consistent with this analysis at the single cell level, the average response shows that shortly after blocking the ATP synthase, the Ca^2+^ signal started to decline (Fig. 2*B*). Within several minutes cytosolic Ca^2+^ returned to basal levels corresponding to the signal before glucose stimulation (Fig. 2, *A* and *B*). Via its effect on Ca^2+^ signaling, inhibition of mitochondrial ATP synthesis also caused a reduction of glucose-stimulated insulin secretion (Fig. 2*E*). These results are consistent with human islet perifusion experiments where azide rapidly lowered insulin secretion (27). We conclude that continuous mitochondrial ATP synthesis is required to maintain Ca^2+^ signaling during metabolism-secretion coupling in both INS-1E and human beta cells.

##### ATP Synthase-dependent and -independent Respiration in Pancreatic Beta Cells

**FIGURE 3.**
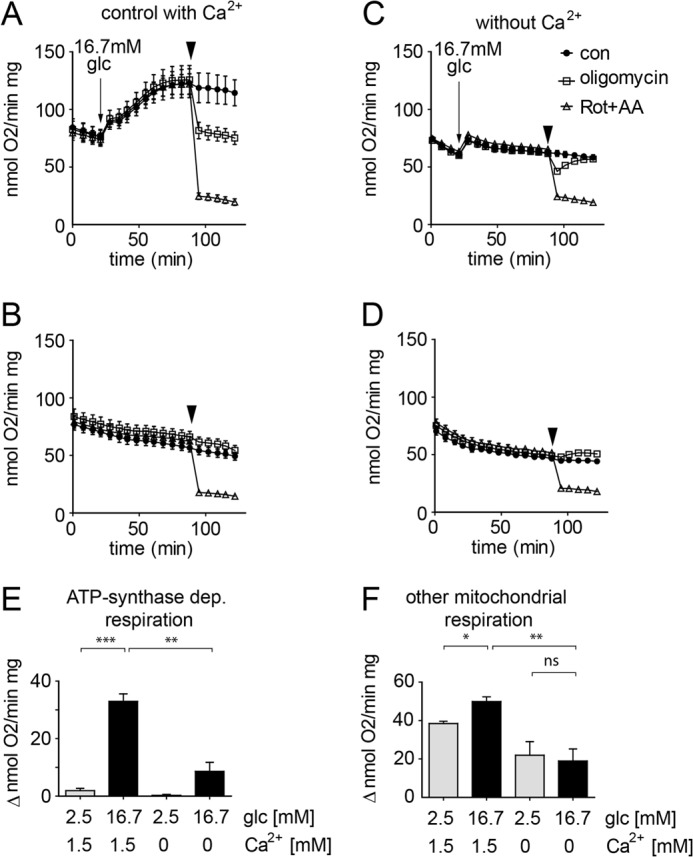
**Glucose- and calcium-induced activation of ATP synthase-dependent respiration in INS-1E cells.** INS-1E cells were assayed in standard KRBH containing 1.5 mm Ca^2+^ (*A* and *B*) or a KRBH lacking Ca^2+^ but including 0.4 mm EGTA (Ca^2+^-free; *C* and *D*). The cells were stimulated by adding glucose to a final concentration of 16.7 mm (*A* and *C*, *arrow*) or maintained continuously under basal conditions 2.5 mm glucose (*B* and *D*). Inhibitors of the respiratory chain were added as indicated (*arrowhead*). For each dataset the following conditions were tested: rotenone (*Rot*; 1 μm) plus antimycin A (*AA*; 1 μg/ml) (*open triangles*), oligomycin (2.5 μg/ml; *open squares*), control (*con*, DMSO; *closed circles*). Quantification of ATP synthase-dependent (*E*) and ATP synthase-independent (*F*) respiration (see “Experimental Procedures”). *A–D*, representative results are shown (*n* = 6, mean ± S.E.). *E* and *F*, quantification of the respiration data (average ± S.E.) under control conditions (*n* = 6) and Ca^2+^-free conditions (*n* = 4). *Glc*, glucose. *, *p* < 0.01; **, *p* < 0.001; ***, *p* < 0.0001; *ns*, not significant.

The respiratory response of INS-1E cells to glucose can be divided into two phases: an immediate acceleration of respiration occurring during the first 5 min and a subsequent slower but continuous acceleration of respiration until a new steady state was reached after 40 min of glucose stimulation (Fig. 3*A*; see also Ref. 5). Total mitochondrial respiration was determined using rotenone and antimycin A, calculating the difference before and after addition. The observed oxygen consumption after the addition of the inhibitors (15% of maximal glucose-induced respiration (Fig. 3*A*)) was considered to be non-mitochondrial and was not further studied. Blocking ATP synthase in glucose-stimulated INS-1E cells using oligomycin caused a rapid but only partial inhibition of respiration. We refer to this fraction as ATP synthase-dependent (oligomycin-sensitive) respiration, which is responsible for about 40% of total mitochondrial respiration in INS-1E cells (Fig. 3*A*). The larger fraction (60%) is mitochondrial respiration not directly linked to ATP synthesis (ATP synthase-independent respiration; see also “Discussion”).

**FIGURE 4.**
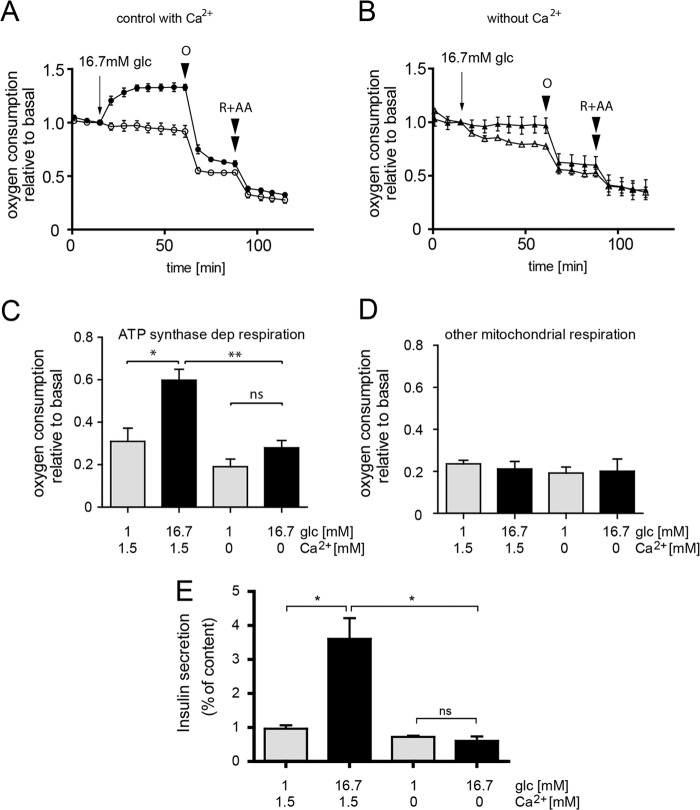
**Glucose- and calcium-mediated activation of ATP synthase-dependent respiration in human islets.** Human islets bound to 804G matrix were incubated in KRBH (*A*) or a KRBH buffer lacking Ca^2+^ supplemented with 0.4 mm EGTA (*B*; Ca^2+^-free). For each condition the mean ± S.E. *n* = 3 from the same donor is shown. Total islet protein varied between wells (4–6 μg). Because of these variations the results are expressed relative to the respiratory rate before glucose stimulation. ATP synthase-dependent (*dep*, *C*) and -independent respiration (*D*) was quantified as described under “Experimental Procedures.” The results are the mean ± S.E. (*n* = 6) obtained from 2 donors. *, *p* < 0.01; **, *p* < 0.001; *ns*, not significant. *R*, rotenone (1 μm); *AA*, antimycin A (1 μg/ml); *O*, oligomycin (2.5 μg/ml); *glc*, glucose. *E*, preventing calcium signaling blocks glucose-induced insulin secretion. Insulin secretion from human islets was determined in KRBH or the same buffer lacking Ca^2+^ in either 1 mm (*gray bars*) or 16.7 mm glucose (*black bars*). Shown is the average ±S.E. *n* = 4 result with islets from a single donor (*, *p* < 0.05).

Respiration of human islets was also enhanced after glucose stimulation (Fig. 4*A*). Human islet data were expressed relative to basal respiration before glucose stimulation. This corrects for the variability of respiration rates between wells due to differences in the number of attached human islets (Fig. 4, *A* and *B*). Glucose-induced respiration increased rather rapidly as a new steady-state of oxygen consumption was reached within 12–18 min (Fig. 4*A*). Oligomycin caused a marked reduction of respiration in glucose-stimulated human islets. Rotenone and antimycin A further reduced respiration as expected. In glucose-stimulated human islets 75 ± 7% of total mitochondrial respiration was oligomycin-sensitive. The observed contribution of ATP synthase activity to respiration was, therefore, much larger than in INS-1E cells (Fig. 3*A*). Consequently, in human islets ATP synthase-independent respiration was smaller (25 ± 7%).

##### Glucose Selectively Activates ATP Synthase-dependent Respiration in Beta Cells

Comparison of oligomycin-sensitive and -insensitive respiration revealed that glucose specifically activated ATP synthase-dependent respiration both in INS-1E cells (Fig. 3, *A* and *B*) and human islets (Fig. 4*A*). In resting INS-1E cells (2.5 mm glucose), oligomycin-sensitive respiration was almost absent (Fig. 3, *B* and *E*). We conclude that the observed glucose-induced acceleration is mainly due to activation of ATP synthase-dependent respiration. Most of this glucose effect is reversed after oligomycin addition (Fig. 3*A*). Consistent with this interpretation, oligomycin-independent respiration was only slightly lower in INS-1E cells maintained in basal conditions (2.5 mm) compared with 16.7 mm glucose (Fig. 3*F*).

In human islets oligomycin also inhibited respiration under basal glucose conditions (Fig. 4*A*). However, after glucose stimulation of human islets ATP synthase-dependent respiration was increased 1.9-fold (Fig. 4, *A* and *C*). The ATP synthase-independent fraction on the other hand was not significantly affected by glucose (Fig. 4, *A* and *D*). We conclude that glucose notably stimulates ATP synthase activity (oligomycin-dependent respiration), having little or no impact on other mitochondrial respiration-dependent processes in INS-1E and human beta cells.

##### Glucose-induced Ca^2+^ Signaling Is Required for ATP Synthase-dependent Respiration

Glucose-induced Ca^2+^ signals are required for the stimulation of mitochondrial respiration (5, 17). We, therefore, tested whether and to what extent Ca^2+^ signaling affects ATP synthase-dependent and -independent respiration. In the absence of extracellular Ca^2+^ (Ca^2+^-free: KRBH without added Ca^2+^ including 0.4 mm EGTA), cytosolic and mitochondrial Ca^2+^ signals in INS-1E cells were completely suppressed and glucose-induced respiration was strongly blunted (Fig. 3*C* and data not shown). In the absence of Ca^2+^ signaling, glucose stimulated respiration initially, but thereafter respiration rates remained close to constant (Fig. 3*C*). Under Ca^2+^-free conditions, ATP synthase (oligomycin)-dependent respiration was 2.5-fold lower than in control glucose-stimulated INS-1E cells (Fig. 3*E*). Unexpectedly, in the absence of Ca^2+^, inhibition of respiration by oligomycin was transient (Fig. 3*C*). The reason for recovery of respiration after oligomycin treatment is not clear. Rotenone plus antimycin A reduced respiration efficiently starting, however, from lower initial values of respiration. As a consequence, ATP synthase-independent respiration was reduced by a factor of 1.8 when Ca^2+^ signaling was prevented (Fig. 3*F*).

In human islets the absence of extracellular Ca^2+^ also had a very strong inhibitory effect on glucose-induced respiration (Fig. 4, *B* and *C*). Oligomycin-sensitive respiration was reduced 2.1-fold in the absence of Ca^2+^ signaling (Fig. 4*C*), whereas oligomycin-insensitive respiration was not significantly affected (Fig. 4*D*). As Ca^2+^ is essential for insulin granule exocytosis, glucose-induced insulin secretion was completely suppressed when Ca^2+^ signaling was prevented (Fig. 4*E*). In the present system, it is therefore not possible to study the impact of Ca^2+^ control of mitochondrial energy metabolism on insulin secretion. In summary, we find that Ca^2+^ signals control ATP synthase-dependent respiration in INS-1E cells and human islets.

##### Mitochondrial ATP Synthesis Is Essential to Maintain the ATP/ADP Ratio

**FIGURE 5.**
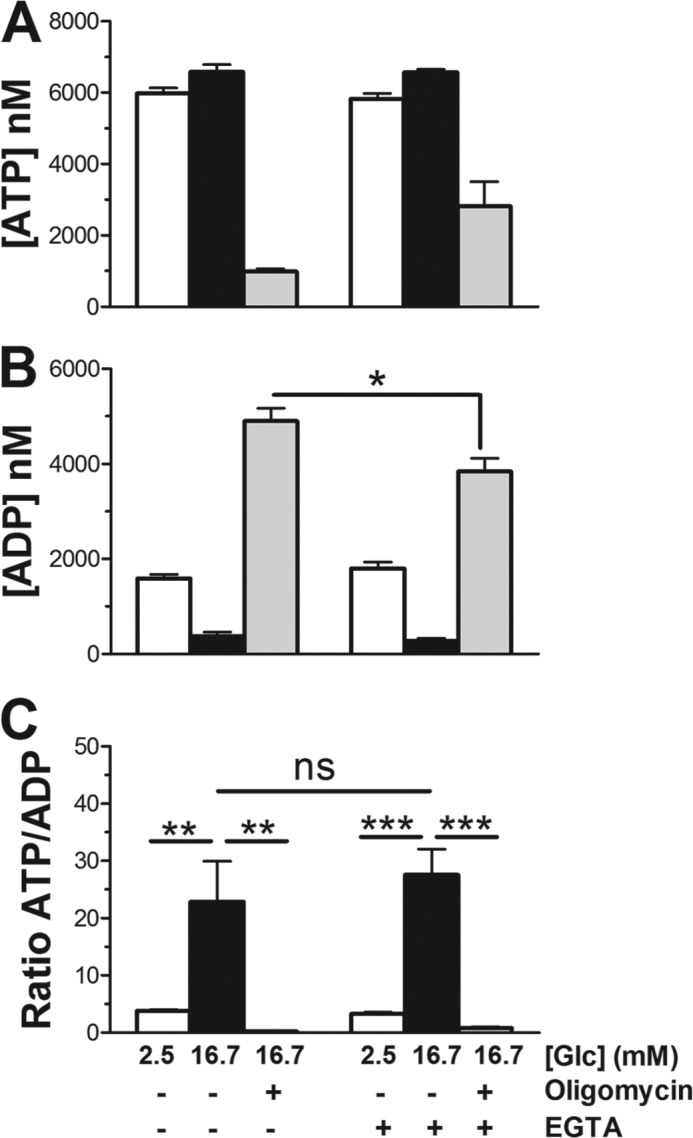
**Glucose and calcium dependent ATP and ADP responses in INS-1E cells.** INS-1E cells were incubated for 30 min in standard KRBH containing 1.5 mm Ca^2+^ or a KRBH lacking Ca^2+^ but including 0.4 mm EGTA at either resting (2.5 mm, *white bars*) or stimulatory (16.7 mm, *black bars*) glucose (*Glc*) concentrations. Where indicated, oligomycin (2.5 μg/ml, *gray bars*) was added during the last 10 min of the 16.7 mm glucose incubation. Glucose-, Ca^2+^-, and oligomycin-dependent changes are shown separately for ATP (*A*) and ADP (*B*) as well as the ATP/ADP ratio (*C*). Shown is the average *n* = 8 ±S.E.; *n* = 2. *, *p* < 0.01; **, *p* < 0.001; ***, *p* < 0.0001; *ns*, not significant.

Activation of mitochondria by glucose-derived pyruvate accelerates mitochondrial ATP synthesis and thereby elevates the ATP/ADP ratio. This increase occurs over a rapid time-course (1–3 min) after glucose addition (28, 29). To test the effect of Ca^2+^ signaling on ATP and ADP changes, we looked at a later time point (30 min; Fig. 5) as the respiration rate became increasingly Ca^2+^ dependent with time (Fig. 3). The change in ATP/ADP ratio is the result of a small increase of the intracellular ATP and a marked reduction of ADP in response to glucose (Fig. 5, *A* and *B*). Blocking mitochondrial ATP synthesis with oligomycin lowered ATP but also increased ADP dramatically. Consequently, the ATP/ADP ratio was >70 times lower than in control glucose-stimulated cells (Fig. 5*C*). We conclude that in INS-1E cells, glycolysis is unable to maintain a physiological ATP/ADP ratio when mitochondrial ATP synthase is blocked. Preventing calcium signaling had no significant effect on the glucose-induced changes in ATP, ADP, or the corresponding ATP/ADP ratio (Fig. 5). These results are consistent with earlier findings with mouse and rat islets (29, 30). In these studies blocking Ca^2+^ signaling even increased the ATP/ADP ratio. Ca^2+^ has been proposed to control both ATP synthesis and ATP consumption, which may explain why preventing Ca^2+^ signaling does not lower the ATP/ADP ratio despite the observed Ca^2+^-dependent reduction in respiratory rate.

##### The Prevailing Glucose Concentration Determines NAD(P)H Levels in Pancreatic Beta Cells

**FIGURE 6.**
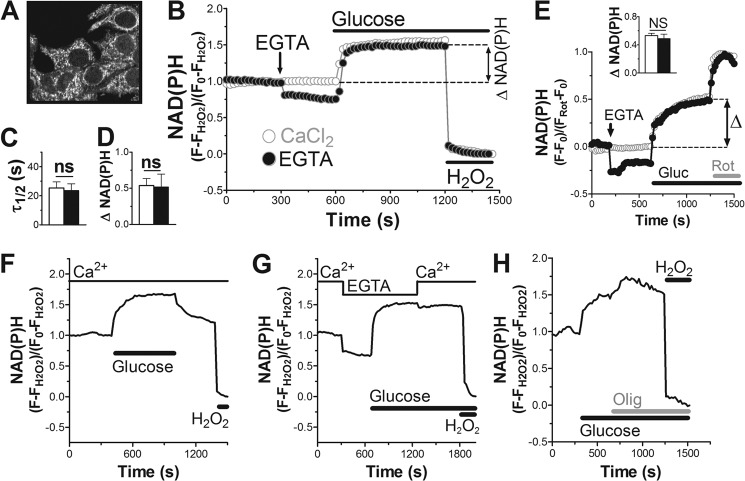
**Effect of calcium signaling on NAD(P)H responses in INS-1E cells.** *A*, NAD(P)H autofluorescence in INS-1E cells at 2.5 mm glucose. *B*, *E*, *F*, *G*, and *H*, kinetics of NAD(P)H fluorescence changes were followed. *B*, the NAD(P)H signal was normalized to the fluorescence measured at basal glucose (set to 1) minus the minimal signal after full oxidation to NAD(P)^+^ using excess H_2_O_2_ (set to 0). Glucose concentrations were raised from 2.5 to 16.7 mm as indicated. EGTA: Ca^2+^-free conditions (KRBH without Ca^2+^ plus 0.4 mm EGTA). *C*, calculated half-time to reach a new steady state of the NAD(P)H signal after glucose stimulation. *D*, net glucose-induced increase of the NAD(P)H signal over basal. Shown is the mean ± S.E. (*n* = 5) for control (*white*) and Ca^2+^-free conditions (*black*). *E*, glucose (*Gluc*)-induced NAD(P)H changes using an alternative calibration method. The responses are defined as increase over basal (set to 0) as a fraction of the maximal NAD(P)H signal obtained after inhibition of complex I with rotenone (*Rot*; 1 μm; set to 1). Shown is the average ±S.E. (*n* = 3) for control (*white*) and Ca^2+^-free conditions (*black*). *Inset*, quantification of the NAD(P)H response. *F–H*, individual measurements of INS-1E cells stimulated with glucose in the presence (*F* and *H*) or absence of Ca^2+^ (*G*). Effect of nutrient removal (*F*), calcium re-addition (*G*), inhibition of respiration with oligomycin (*Olig*; 2.5 μg/ml; *H*) are shown. Data are representative of *n* = 6 (*F*), *n* = 3 (*G*), and *n* = 5 (*H*) experiments. *ns*, not significant.

The ability of Ca^2+^ to control mitochondrial metabolism and respiration has been mainly attributed to the activation of Ca^2+^-regulated dehydrogenases (13). This should accelerate oxidative metabolism associated with the formation of the reduced co-factor NAD(P)H (sum of NADH and NADPH). This reduced form is autofluorescent, whereas the oxidized form NAD(P)^+^ is virtually non-fluorescent. The emission spectra of NADH and NADPH are indistinguishable and, therefore, the two co-factors cannot be measured separately. Nevertheless, the autofluorescence of NAD(P)H can be used as a measure of the NAD(P)H/NAD(P)^+^ redox state and, therefore, indirectly as a read-out of oxidative metabolism (31). NAD(P)H fluorescence was followed on a two-photon confocal microscope. The signal was strongest in particulate or filamentous areas of the cell excluded from the nucleus, most likely reflecting mitochondria (Fig. 6*A*). This is consistent with an earlier study finding 5–8-fold higher concentrations of NAD(P)H in mitochondria compared with the cytosol (31). NAD(P)H signals were followed over time in the INS-1E cell population. The NAD(P)H signal was expressed as relative changes to the signal observed at resting glucose (set to 1) and the minimal fluorescence after oxidation of NAD(P)H using excess hydrogen peroxide (set to 0).

Glucose increased the steady-state NAD(P)H levels consistent with earlier studies (Fig. 6, *B* and *E*; Ref. 31–33). The glucose-induced rise of NAD(P)H was rapid with a half-time of only 25.4 ± 4.2 s to reach maximum values (Fig. 6*C*). The net increase of the NAD(P)H signal varied strongly between different days of the experiment but averaged 53.8 ± 9.7% over basal (Fig. 6*D*). Lowering the glucose concentration rapidly decreased the NAD(P)H autofluorescence (Fig. 6*F*), which returned to basal levels or remained slightly elevated when compared with the signal measured before glucose addition.

**FIGURE 7.**
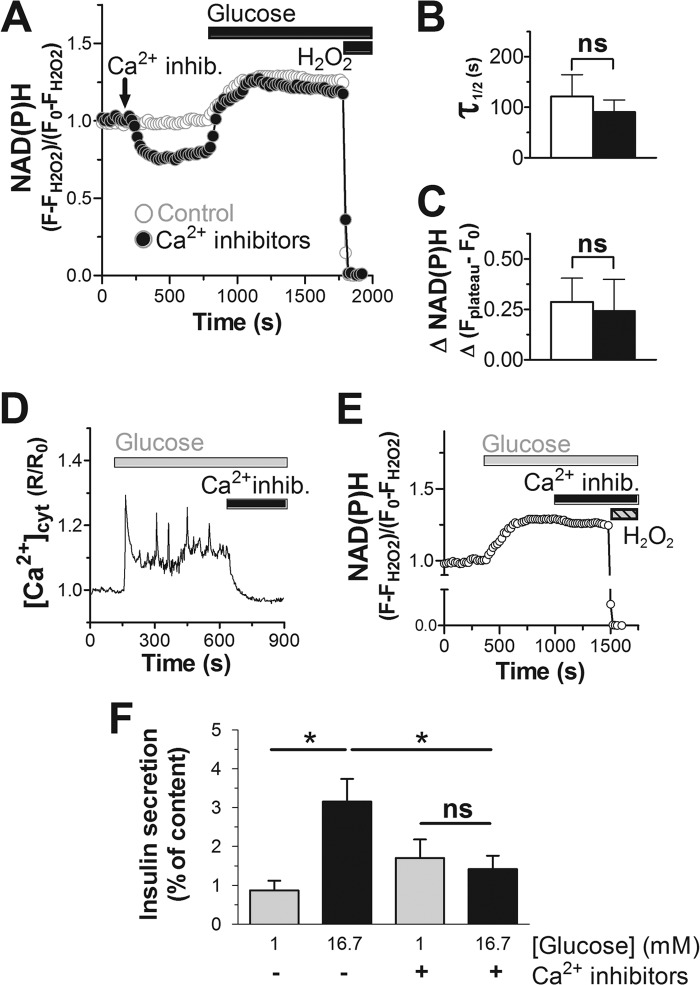
**Effect of calcium signaling on NAD(P)H response and insulin secretion in human islets.** *A*, *B*, *C*, and *E*, the NAD(P)H signal in human islets was followed and quantified as described in Fig. 6. Where indicated the three voltage-dependent Ca^2+^ channel blockers: isradipine (20 μm), ω-agatoxin (400 nm), and NNC 55-0396 2 μm were added (*C*a^2+^ *inhib.*). Glucose stimulation in control KRBH (*white*) or preincubated in the presence of Ca^2+^ channel blockers (*black*). *A*, average glucose responses. *B*, quantification of the half-time for the NAD(P)H signals to reach a new steady state. *C*, net glucose-induced increase of the NAD(P)H autofluorescence. For both conditions the average response from human islets (*n* = 4) originating from 2 donors (*n* = 2) were analyzed. Shown is the mean ± S.E. *D*, cytosolic Ca^2+^ signals were measured after infection of beta cells with an adenovirus carrying YC3.6 as described in the legend to Fig. 2. Human islet cells were stimulated with glucose (16.7 mm). The three Ca^2+^ channel blockers (*Ca*^2+^ *inhib.*) were added as indicated. Data are representative of 25 cells (*n* = 4). *E*, the three Ca^2+^ channel blockers were added once glucose had raised the NAD(P)H signal to an elevated steady-state level. *F*, static insulin secretion from human islets as described under “Experimental Procedures.” Secretion was measured in resting (1 mm; *gray bars*) or stimulatory (16.7 mm; *black bars*) glucose concentrations. Where indicated the three voltage-dependent Ca^2+^ channel blockers were included at the concentrations given above. *, *p* < 0.01; *ns*, not significant.

Similar experiments were performed averaging the NAD(P)H signal over individual islets (Fig. 7). Glucose increased the NAD(P)H autofluorescence rapidly (Fig. 7*A*) with a half-time of 121.2 ± 43.5 s (Fig. 7*B*). The net NAD(P)H fluorescence increase was 28.7 ± 11.7% (Fig. 7*C*) over basal. We conclude that in INS-1E cells and human islets, variations of the glucose concentration are very rapidly translated into changes of the NAD(P)H levels consistent with the role of the beta cell as a nutrient sensor.

Once NAD(P)H signals had reached a steady state after glucose stimulation, the complex I inhibitor rotenone could further elevate NAD(P)H levels (Fig. 6*E*). Complex I mediates the transfer of electrons from NADH, which oxidizes the co-factor to NAD^+^. Blocking complex I, therefore, leads to the accumulation of reduced NADH explaining the increased autofluorescence. A similar response was observed when blocking ATP synthase with oligomycin (Fig. 6*H*). Inhibition of ATP synthase slows upstream respiration and thereby NAD(P)H oxidation. Taken together, these results suggest a balance of NADH production (oxidative metabolism) and NADH oxidation (respiratory chain activity) that determines NAD(P)H steady-state levels.

##### Glucose-induced Ca^2+^ Signaling Does Not Affect Steady-state NAD(P)H Levels in Pancreatic Beta Cells

To assess the effect of Ca^2+^ signals on NAD(P)H, glucose responses were repeated in Ca^2+^-free KRBH containing 0.4 mm EGTA. Removal of Ca^2+^ under basal glucose conditions (2.5 mm) caused an immediate small reduction of the NAD(P)H signal in INS-1E cells (Fig. 6*B*). Although cytosolic Ca^2+^ signals are weak under resting conditions, completely blocking Ca^2+^ influx appears to affect the NAD(P)H steady-state levels consistent with an impact of Ca^2+^ on mitochondrial oxidative metabolism. Surprisingly, when raising glucose in the absence of Ca^2+^, NAD(P)H levels increased as rapidly as under control conditions (23.6 ± 4.7 s; Fig. 6*C*). Furthermore, although the starting level of the NAD(P)H signal was lower, the final steady-state NAD(P)H levels were not significantly different when Ca^2+^ was omitted (Fig. 6, *B* and *D*). Furthermore, re-addition of Ca^2+^ after glucose stimulation in Ca^2+^-free medium did not further increase the NAD(P)H steady-state level (Fig. 6*G*). This lack of calcium dependence was surprising especially given the striking Ca^2+^ effect on respiration (Fig. 3, *A* and *C*). To better quantify the observed NAD(P)H responses to glucose, we used an alternative calibration strategy. As demonstrated in Fig. 6*E*, rotenone, which blocks complex I, increased the NAD(P)H signal to the point where autofluorescence was likely close to maximal. Rotenone was chosen over other inhibitors of the respiratory chain (oligomycin or cyanide) as it gave the most robust and reproducible increase of NAD(P)H signals in INS-1E cells (data not shown). Using this calibration, NAD(P)H responses were expressed as a fraction between basal (set to 0) and maximal signal after rotenone (set to 1). Glucose stimulation of INS-1E cells raised the NAD(P)H signal to 0.53 of maximal. When removing extracellular Ca^2+^ the NAD(P)H signal decreased below basal but reached levels not significantly different from the control after glucose stimulation (0.56). We conclude that steady-state NAD(P)H levels are mainly determined by the prevailing stimulatory glucose concentration with little further influence of Ca^2+^ signaling.

In human islets we failed to assess the effect of Ca^2+^ removal by EGTA. Islets NAD(P)H signals became unstable possibly due to altered binding of the islet to the matrix-coated glass coverslips when Ca^2+^ was chelated. We, therefore, used a combination of Ca^2+^ channel blockers isradipine, ω-agatoxin. and NNC 55-0396, which were shown previously to almost completely prevent voltage-dependent Ca^2+^ uptake in human beta cells (34). The efficacy of these inhibitors to block Ca^2+^ signaling was tested in human islets expressing the calcium probe YC3.6 specifically in beta cells. In combination, isradipine, ω-agatoxin, and NNC 55-0396 ended glucose-induced transients and lowered the cytosolic calcium to basal concentrations before glucose stimulation within 1–5 min after their addition (Fig. 7*D*). By blocking Ca^2+^ influx the three inhibitors also prevented glucose-induced insulin secretion (Fig. 7*F*). In addition, the Ca^2+^ channel blockers reduced the NAD(P)H signal in human islets when incubated under resting glucose conditions (Fig. 7*A*), similar to the effect of removing extracellular calcium from INS-1E cells (Fig. 6*B*). After stimulation with 16.7 mm glucose, NAD(P)H levels increased rapidly whether or not the Ca^2+^ channel inhibitors were present (Fig. 7*B*). Furthermore, the steady-state NAD(P)H signals reached after glucose (16.7 mm) stimulation were similar when the islets were pretreated with the Ca^2+^ channel inhibitors (Fig. 7*C*). We cannot, however, exclude possible smaller differences as net NAD(P)H increases varied strongly between individual islets. To address this issue, we stimulated human islets with glucose first to add the inhibitors of voltage-gated Ca^2+^ channels once the NAD(P)H signal had already reached a new steady state (Fig. 7*E*). Under these conditions, blocking Ca^2+^ influx had little or no effect on the NAD(P)H signal. We conclude that the glucose-induced NAD(P)H levels in beta cells in contrast to the respiratory rate is not strongly influenced by Ca^2+^ signaling.

## DISCUSSION

Mitochondria are able to adjust their ATP synthesis to the energy requirement of the cell. Depending on the biological process, such adjustments must be rapid to avoid a drop of the phosphorylation potential. The mechanisms underlying such control of oxidative phosphorylation remain poorly understood. Early work with isolated mitochondria suggested that mitochondria mainly work on demand, enhancing respiration as a response to an increase in extramitochondrial ADP. In several cell types this control mechanism is not likely of relevance as it was shown that during physiological cell activation the phosphorylation potential does not decrease, and ADP levels are not elevated (1, 8). In the pancreatic beta cell, after nutrient stimulation the phosphorylation potential is even elevated despite an augmented energy demand (7, 28, 29, 35). We have obtained similar results measuring ATP and ADP after glucose stimulation (Fig. 5). Glucose causes a small increase of cellular ATP associated with a marked reduction of ADP. This decrease in ADP was measured after 30 min of glucose stimulation, a time when respiration rates are clearly stimulated (Fig. 5*B*). It is, therefore, unlikely that the ADP concentration is the sole regulator of mitochondrial respiration and ATP synthesis in INS-1E cells.

The observed changes in ATP and ADP are essential for the beta cell to inhibit the *K*_ATP_ channel, which initiates the downstream events leading to insulin secretion. This may be accomplished through the increased provision of nutrient-derived metabolites for mitochondrial metabolism. The term “glucose push” has been coined to describe this initial activation of beta cell mitochondria by glycolysis-derived pyruvate (35). Glucose push provides more pyruvate for oxidative metabolism and may augment the concentration of TCA cycle intermediates via anaplerosis (36, 37). This push mechanism is likely also responsible for the close relationship between NAD(P)H and the glucose concentration. Both raising and lowering the glucose concentration was followed within seconds by respective changes in NAD(P)H autofluorescence (Fig. 6, *B*, *E*, and *F*). These results point to a special characteristic of beta cell energy metabolism. Rapid NADH provision for respiration is able to accelerate respiration over a very short time-course, which is achieved despite decreasing ADP levels. This tight link between glucose concentration and NAD(P)H may allow the beta cell to rapidly increase the ATP/ADP ratio necessary to induce electrical activity. Here we have studied the activation of beta cell mitochondria during glucose stimulation focusing on oxidative metabolism (following the NAD(P)H autofluorescence signal) and ATP synthase (oligomycin)-dependent respiration. Respiration is essential to maintain beta cell secretory activity. Inhibiting respiratory chain complexes blocks glucose-induced insulin secretion (26, 27). Consistent with these findings, we observe that inhibition of respiration either with a combination of rotenone plus antimycin A or specifically by inhibiting ATP synthase with oligomycin rapidly ended cytosolic Ca^2+^ signals in INS-1E cells and human islets. Unlike rotenone and antimycin A, oligomycin hyperpolarizes the inner mitochondrial membrane (38) and only partially inhibits respiration (Figs. 3 and 4). Nevertheless, oligomycin was able to end cytosolic Ca^2+^ transients over a very short time-frame (2–3 min) similar to the combination of the complex I and III inhibitor. Glucose-induced mitochondrial Ca^2+^ signals were also suppressed when blocking ATP synthesis in INS-1E cells. The driving force for Ca^2+^ uptake into mitochondria is normal or even elevated as inhibition of ATP synthase by oligomycin hyperpolarizes the inner mitochondrial membrane (38). Likely, the loss of mitochondrial Ca^2+^ is the secondary consequence of impaired cytosolic Ca^2+^ signaling.

Our results underline the importance of continuous mitochondrial ATP synthesis to maintain beta cell Ca^2+^ signaling. This close correlation suggests that short-lasting fluctuations in mitochondrial activity may contribute to the formation of calcium oscillations and thereby pulsatile insulin secretion.

Respiration is required to pump protons out of the mitochondrial matrix to maintain the mitochondrial electrochemical gradient. Proton entry through the ATP synthase drives ATP synthesis. In most cell types the fraction of ATP synthase-dependent (oligomycin-sensitive) respiration is the main component of mitochondrial respiration (39–42). Our dissection of total mitochondrial respiration in an oligomycin-dependent and -independent component reveals that under resting glucose conditions ATP synthase-dependent respiration is low. These findings are consistent with the hypothesis that under resting conditions mitochondrial ATP synthesis must be prevented to avoid a rise in the cytosolic ATP/ADP ratio that would otherwise result in inappropriate insulin secretion (43).

In agreement with our data, prominent uncoupling has previously been observed in INS-1E cells (40) as well as mouse and human islets (42). Both studies conclude that there is a large proton leak in beta cell mitochondria. This interpretation is consistent with the current literature where uncoupling has become close to synonymous with the futile cycle of protons such as observed during thermogenesis. We would like to stress, however, that the here-observed respiration occurring uncoupled from ATP synthesis is likely to drive other energy consuming processes that are as important to mitochondrial function as ATP synthesis itself. ATP synthase-independent respiration is required to pump protons out of the mitochondrial matrix to maintain the electrochemical gradient balancing the continuous activity of electrogenic transport processes across the inner mitochondrial membrane. Such energy-consuming transport steps are for instance the net export of negatively charged metabolites or the uptake of positively charged ions such as Ca^2+^. The high rates of uncoupling (depolarizing) may also help beta cells to maintain a normal membrane potential despite inhibition of ATP synthase (hyperpolarizing effect) under resting glucose conditions. Uncoupling slows ATP synthesis and, therefore, helps to prevent electrical activity in resting beta cells. Why this is mechanism is further exaggerated in INS-1E cells is not clear.

Glucose stimulation caused a marked acceleration of respiration in INS-1E cells consistent with earlier reports (5, 37). We show that this increase is almost exclusively due to the activation of ATP synthase-dependent respiration. In contrast, oligomycin-insensitive respiration was only slightly increased after glucose stimulation. Qualitatively similar results were observed in human islets. Glucose caused a 2.1-fold increase of ATP synthase-dependent respiration without affecting oligomycin-insensitive respiration (Fig. 4).

After the stimulation of beta cells, both the electrical and chemical component of the mitochondrial membrane potential, increasing the driving force on the ATP synthase (6, 9). This may explain the observed accelerated respiration. Alternatively, mitochondrial respiration may be under kinetic control. The latter mechanism plays an important role for instance in cardiomyocytes by enhancing ATP synthase activity during cell stimulation (44). We speculate that the prevailing glucose concentration affects mitochondrial respiration by a similar mechanism controlling ATP synthase activity or another rate-limiting step of mitochondrial respiration.

Mitochondrial activation depends on mitochondrial matrix Ca^2+^ rises (14, 15). These mitochondrial Ca^2+^signals have been proposed to act mainly through the stimulation of matrix dehydrogenases (13). To address this, we have studied the kinetics of oxidative metabolism via NAD(P)H autofluorescence in INS-1E cells and human islets. Glucose stimulation results in a rapid increase in total NAD(P)H fluorescence. Interestingly, removal of extracellular Ca^2+^, which prevents glucose-induced Ca^2+^ signals, did not affect the rate of the glucose-induced NAD(P)H rise. To block Ca^2+^ signaling in human islets, we used a combination of Ca^2+^ channel blockers (34). In the presence of L-, P/Q-, and T-type Ca^2+^ channel blockers, depolarization-induced Ca^2+^ signaling in human islets beta cells was completely suppressed. In contrast, the rapid glucose-induced rise of NAD(P)H was unaffected. These results are further supported by kinetic data, which show that the NAD(P)H rise precedes the glucose-induced Ca^2+^ signals (Figs. 1, 2, 6, and 7) consistent with earlier findings (32, 33). We propose that the observed increase of the NAD(P)H signal is principally due to the increased provision of glucose-derived pyruvate for mitochondrial metabolism. Preventing Ca^2+^ signaling did, however, cause a small reduction of the steady-state levels of NAD(P)H in resting beta cells consistent with the possibility that Ca^2+^ signals do have an impact on the NAD(P)H redox balance when substrate levels are limiting. Taken together our findings demonstrate that the initial activation of beta cell mitochondria by glucose drives NAD(P)H formation without a need for Ca^2+^ signaling.

In contrast, the glucose-induced respiratory response was almost completely suppressed when Ca^2+^ signals were prevented both in INS-1E cells and human islets (Figs. 3*C* and 4*B*). Also, from a kinetic point of view, NAD(P)H and respiratory responses were clearly distinct. After the rapid glucose-induced response, the NAD(P)H signal remained elevated as long as the stimulatory glucose concentrations was maintained. Respiration augments rapidly early after glucose stimulation and continues to increase at later time points when NAD(P)H has already reached a new steady state.

**FIGURE 8.**
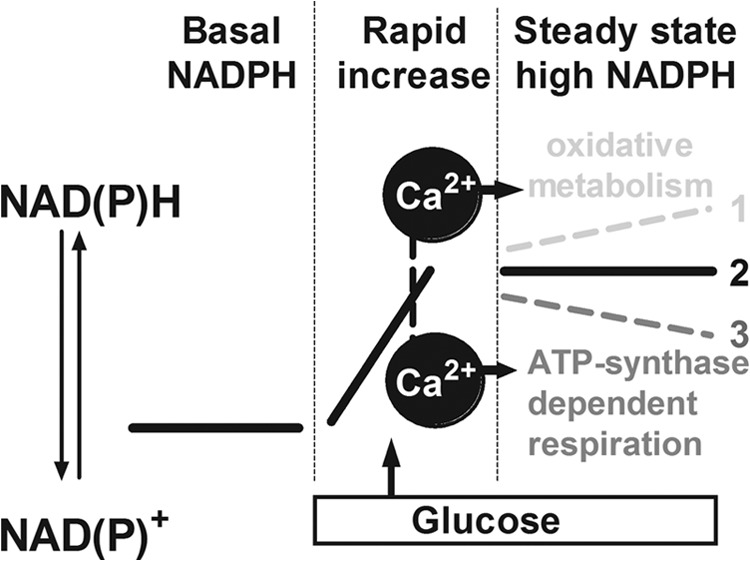
**Proposed model for the coordinated regulation of oxidative metabolism and ATP synthase dependent respiration by Ca^2+^ in pancreatic beta cells.** After glucose stimulation NAD(P)H levels rapidly increase (*1*). Continued selective activation of oxidative metabolism would further increase the NAD(P)H/NAD(P)^+^ ratio (*3*). Activation of ATP synthase-dependent respiration without stimulation of oxidative metabolism should lower the NAD(P)H levels (*2*). Mitochondrial Ca^2+^ signals cause a coordinated activation of oxidative metabolism and ATP synthase-dependent respiration. Rapid establishment of a new NAD(P)H steady state despite continued Ca^2+^-dependent activation of mitochondrial respiration/energy metabolism (experimentally observed in this study) is shown.

Based on these kinetic data we propose a model (Fig. 8) of coordinated regulation of oxidative metabolism (Ca^2+^-sensitive dehydrogenases) and respiration (rate-limiting complex of the respiratory chain or ATP synthase). The initial rapid NAD(P)H increase depends on glucose push. After this early response, Ca^2+^ continuously activates dehydrogenases to maintain NAD(P)H at this elevated level. Although the dehydrogenases produce more NADH per time during this second phase, no further net increase of the NAD(P)H signal was observed. This is due to the accelerated respiratory chain activity, which assures rapid re-oxidation. Such coordinated activation of dehydrogenases and oxidative phosphorylation allows a net increase in respiration without further affecting the NAD(P)H/NAD(P)^+^ ratio. Our population experiments do not exclude the possibility that at the single cell level there are Ca^2+^-dependent NAD(P)H transients as observed in a number of cell types previously (32, 45). In our hands the NAD(P)H signal was not sufficiently strong to perform such single cell analysis.

Fitting with our working model, the NAD(P)H levels observed after glucose stimulation were in an equilibrium that could be shifted to a more oxidized or reduced state. Removal of substrate rapidly lowered the NAD(P)H signal, whereas inhibition of respiration with either rotenone or oligomycin caused a rapid increase of the NAD(P)H autofluorescence. Coordinated regulation of NAD(P)H formation and oxidation implies that Ca^2+^ activates oxidative metabolism as well as respiration. This is in agreement with earlier studies using isolated mitochondria that observed pronounced activation of respiration by Ca^2+^ independent of its effect on oxidative metabolism (21, 46).

The present study finds that ATP synthase-dependent respiration is small in resting beta cells and increases markedly after glucose stimulation. In addition, glucose increases NAD(P)H levels by providing elevated concentrations of the mitochondrial substrate pyruvate. Subsequently, Ca^2+^ signaling linked to cell activation is required to increase ATP synthase-dependent respiration for full activation of beta cell mitochondria. This activation occurs gradually over 12–18 min in human islets during which oxidative metabolism and respiration are increased in a coordinated manner (Fig. 8). As a result, ATP synthase-dependent respiration accelerated markedly without any further change in NAD(P)H. The molecular mechanisms allowing mitochondrial Ca^2+^ signals to stimulate respiration and ATP synthase activity remain to be identified.

## Footnotes

3 The abbreviations used are:

*K*_ATP_: ATP-sensitive potassium channel
INS-1E: rat insulinoma cell line clone 1E
KRBH: Krebs-Ringer bicarbonate Hepes buffer
YC3.6: fluorescent protein yellow cameleon Version 3.6.
